# Three-dimensional Printing in Orthopaedic Surgery: Current Applications and Future Developments

**DOI:** 10.5435/JAAOSGlobal-D-20-00230

**Published:** 2021-04-20

**Authors:** Colleen M. Wixted, Jonathan R. Peterson, Rishin J. Kadakia, Samuel B. Adams

**Affiliations:** From the Department of Orthopaedic Surgery, Duke University, Durham, NC.

## Abstract

Three-dimensional (3D) printing is an exciting form of manufacturing technology that has transformed the way we can treat various medical pathologies. Also known as additive manufacturing, 3D printing fuses materials together in a layer-by-layer fashion to construct a final 3D product. This technology allows flexibility in the design process and enables efficient production of both off-the-shelf and personalized medical products that accommodate patient needs better than traditional manufacturing processes. In the field of orthopaedic surgery, 3D printing implants and instrumentation can be used to address a variety of pathologies that would otherwise be challenging to manage with products made from traditional subtractive manufacturing. Furthermore, 3D bioprinting has significantly impacted bone and cartilage restoration procedures and has the potential to completely transform how we treat patients with debilitating musculoskeletal injuries. Although costs can be high, as technology advances, the economics of 3D printing will improve, especially as the benefits of this technology have clearly been demonstrated in both orthopaedic surgery and medicine as a whole. This review outlines the basics of 3D printing technology and its current applications in orthopaedic surgery and ends with a brief summary of 3D bioprinting and its potential future impact.

Three-dimensional (3D) printing (additive manufacturing) has revolutionized the design theory and manufacturing processes behind a wide range of products in all major industries, providing substantial opportunity for easy prototyping, small production runs with opportunity for real-time refinement, and customizability. Creating geometrically complex and heavily detailed designs and even one-off manufacturing that would not be feasible with traditional production methods has been made possible with this powerful technology. In addition, traditional manufacturing tends to require a central manufacturing site with space to store large inventories. On-demand manufacturing, made possible with 3D printing, has changed this workflow and eliminated the need for a large production and storage space. The technology has become an integral component to commercial manufacturing and made its way into personal homes with the advent of desktop 3D printers. With compatible software and appropriate materials, consumers can witness the transformation from starting material to finished product of their own designs.

Within the field of orthopaedic surgery, 3D printing has impacted patient care and education in several orthopaedic subspecialties.^1-3^ Three-dimensional printed anatomic models are commonly used in preoperative planning and have become a useful educational tool for patient instruction and trainee teaching. For many orthopaedic procedures, including arthroplasty and complex reconstructions, the use of 3D-printed patient-specific instrumentation (PSI) has become commonplace. The excitement around 3D printing continues to build as the fusion of 3D printing and biomedical science has shown early promise. This review article summarizes the fundamentals of 3D printing, discusses its utility within orthopaedic surgery, and highlights its potential future impact.

## Basics of Three-dimensional Printing in Medicine

**Figure 1:**
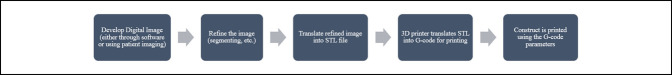
Basic steps of three-dimensional (3D) printing for medical applications. STL = standard triangle language.

Two main types of product manufacturing exist: additive and subtractive. Additive manufacturing fuses successive layers of solids, liquids, or powders to generate the finished product.^4,5^ In contrast, in subtractive manufacturing, the beginning material is cut, milled, or molded from a base product to create the final structure. Various methods of 3D printing exist, but each involves a common stepwise process (Figure 1).

First, a digital representation of the end product is generated through a de novo design or by processing cross-sectional imaging from CT and/or MRI scans saved in the digital imaging and communications in medicine format. This approach enables software to refine these images in the segmentation process to precisely define the shape of the object to be printed with regions of interest, which differentiate between tissues and surrounding anatomical structures.^6,7^ The contours of segmented regions of interest are computationally transformed into an standard triangle language file. In 2011, the additive manufacturing file was approved by the American Society for Testing and Materials, allowing users to integrate additional features of the 3D-printed object into the design (eg, surface texture, color, and material properties).^6^

The next step translates the standard triangle language or additive manufacturing file into a code, typically the G-code, which enables the printer to transform the digitally supplied coordinates of the file into a sequence of two-dimensional cross-sections. These cross-sections are essential as they form the base of each layer, which the printer fuses together to create the final 3D object.^8^

**Figure 2:**
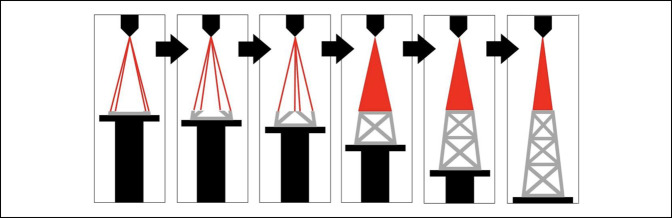
Steps of powder bed fusion from left to right. A layer of titanium powder (gray) is deposited on the printbed. A thermal energy source uses a beam of energy (red) to selectively fuse titanium powder according to data in the design file. The printbed lowers, and a new layer of titanium powder is deposited. The process repeats until the object or objects are completely printed.

**Figure 3:**
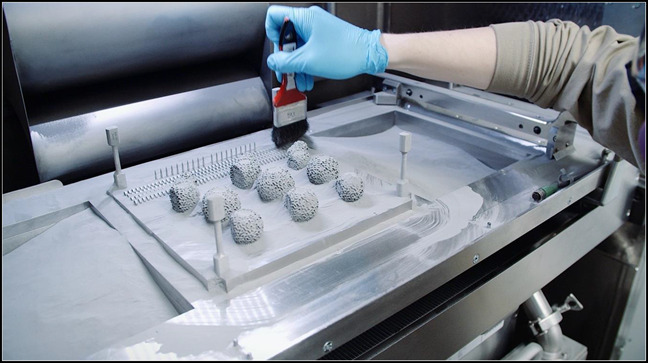
Finished three-dimensional printed implants. The extra titanium powder is brushed away and can be reused.

Once the final product is ready for printing, several methods from which to choose are available, which include material extrusion, material jetting, binder jetting, powder bed fusion, directed energy deposition, stereolithography, sheet lamination, and vat polymerization. Material extrusion, or fused deposition modeling, has become one of the most common printing methods and uses solid-based starting materials. In this process, tiny beads or streams of material exit an extruder in a heated liquid or semiliquid form that is rapidly cooled, forming a hardened layer.^9^ For metal-based products, powder bed fusion-based methods have proven to be successful and are commonly used for orthopaedic implants. A thin layer of powder is deposited on the building platform of the printer, where a thermal energy source, either laser or electron beam, fuses the appropriate region as indicated by the original design. This process is repeated for each layer or the slice of the structure until each has been fused properly, resulting in the desired final product (Figures 2 and 3).

Traditional (subtractive) manufacturing relies on a base product that is milled or cut away to obtain the desired structure, resulting in waste and production of scrap. In contrast, additive manufacturing results in decreased amounts of raw material waste with reported rates less than 5%.^10,11^ This advantage has made additive manufacturing a popular and efficient alternative; in addition, customized products are typically more expensive and time-consuming when traditional methods are used.^12,13^ Although 3D printing is accompanied by its own set of limiting factors, its growing popularity and expansion across industries has substantially decreased costs, increased access, and led to increasing applications in several industries, including medicine.^9,12,14^

## Orthopaedic Applications

An overview of cited literature is provided in Table 1.

**Table 1:** Current Literature on the Applications of Three-dimensional Printing in Orthopaedic Surgery

Factor	Anatomic Models	Noncustom Implants	Patient-specific Instrumentation	Custom Implants	Prosthetics
Education	Kim et al^22^ Bizzotto et al^16^ Zhang et al^72^				
Foot and ankle		Hodsden^30^		Dekker et al^45^	Wojciechowski et al^24^ Xu et al^25^ Xu et al^70^
Joints		Trauner^28^ Wan et al^29^	Kwon et al^35^ Stone et al^36^ Schwarzkopf et al^33^ Attard et al^37^ Leon-Munoz et al^38^	Culler et al^58^ Schwarzkopf et al^59^ Li et al^10^	
Oncology				Ren et al^50^ Xiao et al^51^ Zhang et al^52^ Papagelopoulos et al^53^ Papagelopoulos et al^54^ Imanishi^55^ Wei et al^56^	
Spine		Serra^31^ Mokawem et al^32^		Burnard et al^60^	
Sports					
Trauma	Tetsworth^4^ Michalski^15^ Bizzotto et al^16^ Kang et al^17^ Belien et al^18^ Jeong et al^19^ Kim et al^21^ Chung et al^20^			Nwankwo et al^47^ Tracey et al^48^	

### Anatomic Models

Three-dimensional printed anatomic models are useful both for preoperative planning of complex cases and for teaching purposes. Surgeons can see and feel what they will encounter in the operating room with an accurate representation of the anatomy in 3D space.^15,16^ When more than 100 orthopaedic surgeons were asked to choose a locking plate for a complex tibial fracture after looking at radiograph and CT imaging or a 3D-printed model, surgeons classified as inexperienced, having operated on fewer than 15 similar fractures, changed their preoperative plan over 70% of the time after using the 3D model.^17^ Although experienced surgeons did not change their selection as frequently, more than 70% supported the use of 3D models in their practice if they were available.^17^ In addition to aiding in hardware selection, 3D models allow for prebending of selected plates before surgery. This technique permits the plate to fit the individual anatomy of patients to facilitate accurate reduction and has shown promise in the treatment of clavicle fractures.^18,19^

Three-dimensional printed anatomic models have been used in the mirror imaging technique, in which models of the contralateral uninjured side are printed and used in preoperative planning. Surgeons can use the fractured 3D model to simulate their reduction technique and use the uninjured 3D model to optimize plate selection. This technique has been implemented for clavicle fractures, calcaneal fractures, pilon fractures, and ankle fractures with excellent results.^20,21^

Three-dimensional printed models can be instrumental in medical education. Resident surgeons can develop their technical skills with realistic 3D patient models that illustrate pathologies frequently encountered in the operating room. Trainees who were surveyed regarding the clinical utility of 3D-printed models when planning their surgical approach for percutaneous screw fixation of a posterior column fracture were overall very satisfied, stating that the models deepened their understanding of regional anatomy and the surgical technique.^22^ Patient education has been augmented with 3D-printed anatomic models and may lead to improved patient perioperative understanding and compliance.^16^

Despite the growing interest in and use of 3D-printed anatomic models, they are not currently reimbursed by third-party payers; however, the use of these models leads to significantly shorter operating times. At a mean of $62 operating room time per minute, net savings range from $19,384 to $129,589 and $77,536 to $518,358 for low and high utilization rates, respectively.^23^ Even at low volumes, approximately 63 models per year, estimated cost savings could potentially cover the costs to maintain a 3D printing laboratory.^23^

### Prosthetics and Orthotics

Most braces and orthotics are available only in a limited number of sizes and are designed to fit a large fraction of the population. Although fully customizable prosthetics have proven to be effective, the manufacturing process is complex and adds to the overall cost and time required to make these prosthetics.

**Figure 4:**
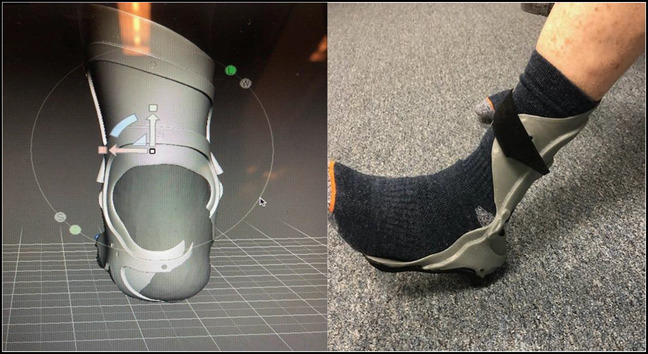
Designing of ankle-foot orthosis (left). The ankle-foot orthosis being fitted to the patient (right).

In contrast, 3D printing has revamped the design and production of ankle-foot orthoses (AFOs). Traditionally, AFOs are made from plaster castings of a patient's lower extremities, a labor-intensive and costly process that leads to problems with fit, comfort, and the overall design and appearance.^24^ Three-dimensional printing has simplified the manufacturing process while facilitating a design that integrates the unique biomechanical metrics of each individual. For patients with plantar fasciitis, these 3D-printed AFOs have shown favorable outcomes.^25^ An example of a 3D-printed AFO is shown in Figure 4.

Three-dimensional printing has reached patients' homes with the introduction of the desktop 3D printer. The straightforward manufacturing process has enabled amputees to print their own prosthetics.^26,27^ This mode of prosthetic production could be an affordable and accessible solution for a large number of patients. However, no FDA approval currently exists for these 3D-printed devices, and regulation on their distribution is lacking.^27^ A systematic review evaluating the clinical efficacy of 3D-printed upper limb prosthetics concluded that all studies meeting inclusion criteria failed to compare the 3D-printed prosthetics with currently available products or production methods, and only one article had sufficient power to detect clinically significant effects.^26^ These studies did report favorable outcomes from the patient perspective and encourage the use of 3D printing as a new avenue for customized prosthetic development.

### New Noncustom Implants

Three-dimensional printing technology can be used to produce orthopaedic implants that are not customized. Several new implant types for hip and knee arthroplasty have entered the market as a result of the streamlined 3D printing production process. Three-dimensional printed acetabular cups are thinner and less expensive than traditionally manufactured cups.^28^ A recently published study on a small group of patients who underwent revision of an acetabular defect with a 3D-printed acetabular cup reported improved stability, better hip scores, and decreased pain.^29^ The increased porosity and homogenous aperture of the 3D-printed cup have been hypothesized to facilitate bone growth better than traditionally manufactured cups.^29^ In a similar fashion, 3D printing has led to the development of porous metal implants for foot and ankle arthrodesis. These implants serve as an alternative to traditional plates, screws, and staples, providing sufficient structural support and improved surface for biological incorporation.^30^

Additive manufacturing has provided new strategies to refine the shape, rigidity, and material of new, innovative cage prototypes of interbody cages for spine surgery. The goal was to create a product that more accurately reflects properties of native bone. Preliminary studies evaluating mechanical properties of 3D-printed intervertebral fusion cages have found that they closely mimic the compressive modulus of trabecular bone.^31^ After implantation of a 3D-printed lamellar titanium cage packed with bone graft, a particular study found a 98.9% arthrodesis rate at 1 year in 93 patients undergoing spinal fusion.^32^

### Patient-specific Instrumentation

Customized surgical guides for orthopaedic surgery have been manufactured with the aid of 3D printing technology.^33,34^ Although it has been proposed that PSI reduces operative time and improves alignment, studies of total knee arthroplasty (TKA) demonstrated mixed results.^35,36^ To preserve a high standard of patient care with a growing case load, an in-depth investigation into the economic efficiency of PSI is valuable. A randomized controlled trial of TKA analyzed the efficiency of conventional instrumentation, PSI, and single-use instrumentation. Cases were classified into four groups: conventional/reusable, patient-specific/reusable, conventional/single-use, and patient-specific/single-use instrumentation. Patient-specific/reusable instrumentation was the most expensive but demonstrated good outcomes: shorter surgery times, less blood loss, shorter length of stay, and higher Oxford Knee Scores 6 weeks postoperatively.^37^ Single-use instrumentation prevented sterilization complications and avoided excess costs related to instrumentation but had no effect on efficiency.^37^ Whether PSI in primary TKA has a definitive advantage is still unclear; however, a recent review found that most publications on this topic do not claim a significant advantage of its use, yet they did not identify a completely negative impact on the accuracy of the procedure either.^38^

Three-dimensional printed patient-specific cutting jigs enable precise and accurate preoperative planning in complex cases of deformity. Correcting angular and rotational deformity can be challenging and requires intense preoperative planning. Clinical outcomes often depend on the accuracy of correction. Three-dimensional printed cutting and locking guides allow for extensive preoperative planning to maximize intraoperative success. Improvements in accuracy have been noted in medial closing wedge distal femoral osteotomy for valgus knee malalignment and lateral compartment disease.^39^ Patients with acetabular fractures, which are difficult to assess and treat because of the complex anatomy of the acetabulum, have more precise screw and plate placement because of 3D-printed guiding templates created from CT scans of the pelvis.^40^

The issues that can arise with PSI merit additional discussion. Three-dimensional printed PSI is designed to control the cutting and reduction according to the surgical plan, which in theory should improve the predictability of the procedure. Although the utility of these guides should not be understated, they remain technically demanding procedures. A small series of patients with uniplanar, biplanar, or triplanar malunion of the long bones underwent corrective osteotomies with 3D-printed patient-specific guides. For malunions of the lower extremities, almost all clinical measurements of the femur and tibia demonstrated an undercorrection postoperatively. Patients with malunions of the humerus had axial and sagittal correction rates that differed substantially between planned and achieved measurements.^41^ Overall, the authors summarized their experience with 3D-printed PSI and concluded the following: careful examination of planned guide positioning is imperative for complete correction intraoperatively, use of predrilled screw holes does not guarantee accurate screw position, translation of bone fragments over osteotomy planes in the case of an oblique osteotomy warrants careful evaluation, and estimation of the depth of osteotomy is difficult and can lead to cartilage damage.^41^

### Patient-specific Custom Implants

Although standard implants are made to fit most of the general population, a personalized fit is required in cases with variations in anatomy and cases in which no already produced implant would suffice (eg, severe bone loss for trauma, cancer, and infection). Custom implants are arguably the most ground-breaking aspect of 3D printing for orthopaedic surgery; surgeons can now design and implant custom devices. Although this technology has the potential to revolutionize patient care, we must also exercise caution and obey the mantra “just because you can, doesn't mean you should.”

Understanding the indications and contraindications of using custom implants is important. The primary indication is cases in which currently available implants will not adequately treat the patient. General contraindications include active infection, vascular compromise, poor bone quality, and a poor soft tissue envelope. Further contraindications are region and subspecialty specific.

**Figure 5:**
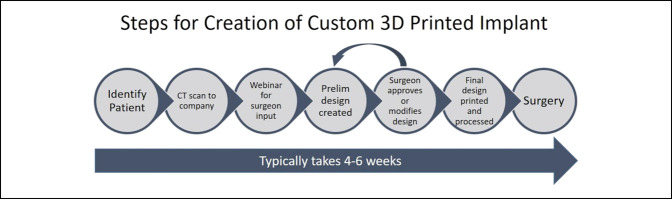
Design process of a custom three-dimensional printed implant.

Once a patient has been identified for a 3D-printed custom implant, a prescription form is required to describe the pathology and document the unique need for a custom implant. In addition, preoperative imaging is needed. Typically, a CT scan and radiographs are submitted for the engineering team to create a 3D model of the patient's anatomy. Next, the surgeon and company representatives meet to discuss the patient's problem and implant design considerations, typically via a webinar. The surgeon should be ready to describe the goals and function of the implant. From the initial design meeting, one or more designs are created, which the surgeon approves or modifies. After the final design is approved by the engineering team and the primary surgeon, the process of fabricating the implant via 3D printing begins. This process is summarized in Figure 5.

Custom implants are granted FDA approval through Section 520(b) of the Food, Drug, and Cosmetic Act.^42^ Several terms must apply for these implants to fall within this category of custom devices. First, each implant is designed for a specific patient at the prescription of a physician. Furthermore, the anatomy or pathology indicated must necessitate the use of a custom implant and cannot be treated with an implant already commercially available in the United States. Thus, the custom implants apply only on a case-by-case basis to manage unique and patient-specific pathology.

**Figure 6:**
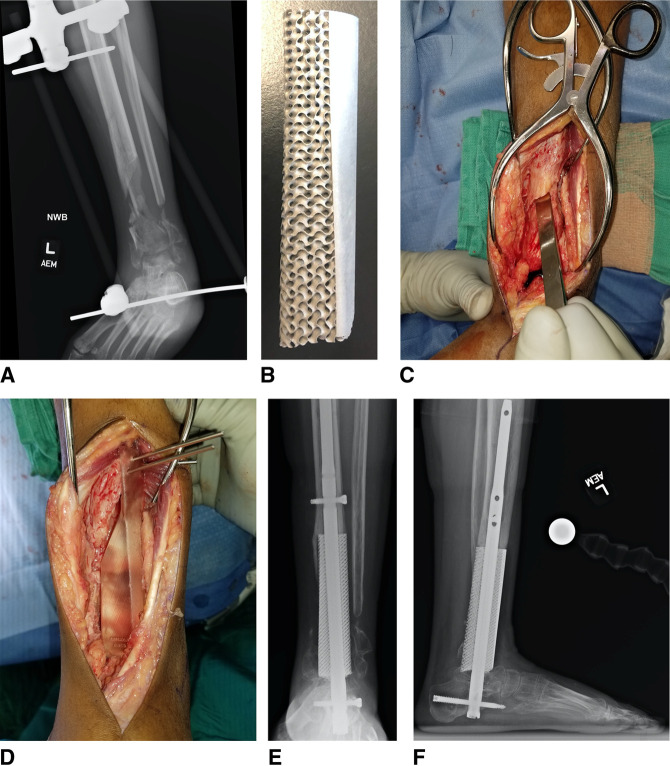
**A,** Anterior-posterior (AP) radiograph of the left leg of a 22-year-old woman who was injured in a motor vehicle collision. She sustained an open tibia and fibula fracture. Bone was lost at the scene, leaving a large bony defect. **B,** Custom three-dimensional (3D) printed implant designed to fill the bony defect. **C,** Intraoperative image showing the distal tibia fracture and bone loss. **D,** Three-dimensional printed anatomic spacer block to assess alignment and length and to perform intramedullary reaming. **E,** AP radiograph demonstrating successful implantation of the 3D-printed implant. **F,** Lateral radiograph demonstrating successful implantation of the 3D-printed implant.

Three-dimensional printing has played a major role in the production of these patient-specific implants with growing evidence of its clinical success. An example of a patient receiving a custom 3D-printed implant for a large bony defect sustained in a motor vehicle collision is shown in Figure 6. For large bone defects arising from traumatic bone loss, deformities, and nonunions, currently used strategies include allograft bone reconstruction, vascularized bone grafts, noncustom metal augments, and bone transport.^43^ Each of these treatment modalities has its own drawbacks (Table 2), with the literature showing mixed clinical outcomes.

**Table 2:** Limitations of Current Methods to Treat Large Bony Defects

Allograft	Autograft	Vascularized Autograft	Non–Three-dimensional Printed Metal Augments	Bone Transport
Limited size and shape	Limited size and shape	Limited size and shape	Limited size and shape	Patient tolerance of the external fixator
Collapse of dead bone	Limited biologic activity with increasing age and comorbidities	Unable to be performed in compromised patients (eg, smokers)		Pin site infection

Studies of 3D-printed patient-specific implants have demonstrated early promising results in cases of segmental bone defects.^44^ Fifteen patients underwent treatment with a 3D-printed custom implant for severe bone loss, deformity correction, and/or arthrodesis procedures of the foot and ankle and demonstrated success with only two failures reported (one nonunion and one infection).^45^ Custom 3D-printed sphere implants have been safely used for patients undergoing tibiotalocalcaneal arthrodesis with more patients achieving successful fusion compared with patients receiving femoral head allografts.^46^ Recently, Nwankwo et al^47^ reported a 5-year follow-up of a distal tibia 3D-printed cage used for severe bone loss secondary to trauma, which is currently the longest known follow-up of a 3D-printed custom implant. In addition, 3D-printed custom talar prostheses have been increasingly used in the treatment of talar osteonecrosis. Total talus replacement with a 3D implant restores talar height and talar tilt while preserving the range of motion and normal alignment in unaffected joints.^48,49^ In addition, custom 3D-printed implants have been commonly used after the excision of primary and metastatic bone lesions.^50,51,52,52,53,54,55,56^

Patient-specific custom implants have become desirable alternatives to standard implants in TKA and total hip arthroplasty procedures. Custom implants have been shown to provide improved rotational alignment and tibial fit.^57^ Furthermore, compared with those treated with off-the-shelf implants, patients with custom implants have lost less blood, reported fewer adverse events, and were less likely to be discharged to a rehabilitation or acute care facility.^58,59^

Spine surgery has implemented 3D-printed patient-specific implants for complex spinal pathology with significant structural deformities, as in cases of neoplasia, degenerative disease, infection, trauma, and congenital anomalies. A systematic review evaluating the efficacy and safety of 3D-printed implants for spine surgery compared with off-the-shelf implants found that all included studies that reported clinical outcomes showed significant postoperative improvements.^60^ Several authors of articles included in this review commented on the significant commitment that 3D-printed spine implants require—there exist a large amount of preoperative work and requirements for specialized design, manufacturing equipment, and personnel that should be recognized before use.^60^ Surgeon involvement in the process is paramount, and they must work closely with the 3D printing company to design the implant. In addition to these intensive time and labor requirements, customized implants can accrue significant financial costs. A careful discussion with the hospital, patient, and insurance company regarding the financial burden of using custom implants is critical.

## Bioprinting/Four-dimensional Printing

Three-dimensional printing technology has advanced rapidly, and several researchers are working on technology to print customized human tissue and organs. Known officially as 3D bioprinting, this process distributes cells, biomaterials, and supporting biological factors in a layer-by-layer fashion to form living tissues and organ analogs.^61,62^ To make this possible, the medium for printing is composed of inert material that can support live cells. Examples include hydrogels, microcarriers, tissue spheroids, cell pellet, tissue strands, and decellularized matrix components. The optimal medium must be stable, nontoxic, nonimmunogenic, biocompatible, and allow for cellular survival and proliferation.^62,63^ The metamorphosis to human tissue or organ analog is accomplished via droplet, extrusion, or laser-based methods. This process facilitates precise control of the microarchitecture and macroarchitecture of the final product, both of which are essential to the function of biologic tissues. These 3D products still face many challenges: growing the correct number of functioning cells, reaching the appropriate cell density, and retaining viability throughout the printing process, but its future potential could revolutionize regenerative medicine.^64^

### Cartilage Bioprinting

Surgical management for articular cartilage injuries vary depending on the location, size of the lesions, and patient factors.^65-67^ Although appropriately selected and performed surgical options can have good clinical results, they fail to fully restore the damaged cartilage tissue. Most restorative techniques create a form of functional cartilage; however, it is not the same as healthy articular cartilage at a molecular level.^66,68^ Three-dimensional bioprinting presents an alternative solution as the ability to print native cartilage would be groundbreaking in the management of cartilage defects and arthritis. Although most works on 3D bioprinting cartilage have been performed in vitro, in vivo animal studies have shown promise. Three-dimensional cartilage cells were implanted into rabbit models of cartilage defects and were found to demonstrate early cartilage formation and osteochondral integration.^69,70^ Moreover, a recent systematic review evaluating the published data surrounding bioprinted articular cartilage endorsed the potential of this technology for use in humans.^71^

### Bone Bioprinting

Bone possesses a unique set of mechanical and structural properties that is challenging to recreate artificially, and advances in 3D bioprinting could aid in bone formation and growth. Scaffolds are an essential technology for both bone tissue engineering and regenerative medicine as they provide the substrate where cells can attach, proliferate, and differentiate into bone. Important characteristics to consider are biocompatibility, biodegradability, microstructure, and osteoconductivity. With the advent of 3D printing, it has become easier to control the microstructure, which is critical to cell viability and osseous ingrowth.^72^ Furthermore, the material of the scaffold is integral to maintaining cell viability and facilitating osteogenic differentiation.^61^

Calcium phosphate is one of the most commonly used materials for 3D-printed bone scaffolds and has gained attention for its superior biodegradability. Ogose et al^73^ reported that nearly all of the tricalcium phosphate implanted in bone defects after the excision of bone tumors were absorbed and replaced with newly formed bone, whereas HA did not demonstrate any biodegradation.

The bioprinting process is a threat to the viability of the cells because they must endure the pressure and shear stress of the printing process and then manage to migrate and proliferate appropriately while receiving sufficient blood supply.^74^ For this reason, long-term viability of bioprinted cells has become a major concern, yet 3D bioprinting remains an exciting new technology that has countless applications for orthopaedic surgeons.

### Four-dimensional Printing

Four-dimensional (4D) printing uses the same set of technologies as 3D printing but adds in one more dimension by allowing the printed part to change shape over time in response to a specific environment. Although similar to 3D bioprinting, this process uses smart materials to create self-reconfigurable proteins, tissue, and organs.^75^ Four-dimensional printed objects can self-repair or self-assemble by changing or reshaping their parts in response to varying environmental conditions (eg, temperature, pH, magnetic field, and solvent interaction). For example, photothermal-responsive shape memory bone tissue engineering scaffolds were constructed and exposed to near-infrared radiation before implantation so that they could be easily molded and configured into a bony defect. After implantation, the temperature rapidly decreased to 37 degrees Celsius, at which temperature the scaffold displayed mechanical properties analogous to those of cancellous bone. This method was successful in treating irregularly sized rat cranial bone defects with improved new bone formation observed.^76^

## Summary

Three-dimensional printing is an exciting technology that is pervasive in every major industry. This rapidly advancing field has created access to almost limitless 3D structures created from a growing variety of materials, including metals, plastics, and even living cells. The benefits of 3D printing include extreme flexibility to customize shapes, increased intricacy/complexity of manufactured products, elimination of assembly steps, and waste and inventory reduction.

In general, disadvantages of 3D printing are similar to those of any new technology and include cost and lack of data, both of which are important to the economically strapped and litigious medical field regarding custom medical implants. However, patient-specific 3D-printed implants offer a new technology to successfully treat a variety of pathologies in orthopaedic surgery. Three-dimensional printing technology will continue to advance and improve patient care and satisfaction.
